# The Fisherman

**DOI:** 10.14797/mdcvj.1062

**Published:** 2021-12-15

**Authors:** James B. Young

**Affiliations:** 1 Executive Director of Academic Affairs, Cleveland Clinic; Professor of Medicine, Cleveland Clinic, Lerner College of Medicine of Case Western Reserve University, Cleveland, Ohio, US

## Abstract

Although I can see him still—
The freckled man who goes
To a gray place on a hill
In gray Connemara clothes
At dawn to cast his flies—
It’s long since I began
To call up to the eyes
This wise and simple man.
All day I’d looked in the face
What I had hoped it would be
To write for my own race
And the reality:
The living men that I hate,
The dead man that I loved,
The craven man in his seat,
The insolent unreproved—
And no knave brought to book
Who has won a drunken cheer—
The witty man and his joke
Aimed at the commonest ear,
The clever man who cries
The catch cries of the clown,
The beating down of the wise
And great Art beaten down.

Maybe a twelve-month since
Suddenly I began,
In scorn of this audience,
Imagining a man,
And his sun-freckled face
And gray Connemara cloth,
Climbing up to a place
Where stone is dark with froth,
And the down turn of his wrist
When the flies drop in the stream—
A man who does not exist,
A man who is but a dream;
And cried, “Before I am old
I shall have written him one
Poem maybe as cold
And passionate as dawn.”

William Butler Yeats

**Poetry Magazine, Vol.7, No. 5, February 1916**

Poetry Foundation

https://www.poetryfoundation.org/poetrymagazine/poems/13324/the-fisherman

This poem is in the public domain.

## Aequanimitas and Trout Fishing

This edition of *Poet’s Pen* was chosen to honor Dr. Craig M. Pratt, who sadly passed away this year. As many know, Craig was a passionate fisherman—particularly of trout, and generally in the many lakes of the Northern and Eastern Sierra Nevada slopes and wild rivers of California, Colorado, and Utah. Not so much a fly fisherman, as reflected in Yeats’ great poem, “The Fisherman,” Craig instead focused on bait and lures during my trips with him—most of the time catching lunch or dinner, which I could never do. Yeats’ poem is a fitting tribute to a man who had a deep love of nature and the stillness and equanimity it afforded.

Fishing has long been a focus of the written arts, with generations of writers and poets celebrating the chance to escape into nature. Craig knew of Isaac Walton’s mid-17th-century classic “The Complete Angler” and also that Sir William Osler’s Coat of Arms and Crest (granted in 1911 when he was knighted) featured three Cornish pilchards (sardines) in tribute to his sea-going ancestors and son, Revere, also a passionate fisherman. “Aequanimitas” is, arguably, William Osler’s most famous essay. It originated from a valedictory address to University of Pennsylvania medical school graduates. The essay focused on imperturbability: “coolness and presence of mind under all circumstances, calmness amid storm, clearness of judgement in moments of grave peril….” This was Craig, the master clinician educator, and Craig, the fisherman in the wilderness. He was, in all ways, resilient. Resilience is informed by equanimity and born of “stillness” that can calm and even heal. Stillness can be found in many ways, but trout fishing with Craig in Thousand Island Lake at 10,000 feet (which is on the John Muir Trail, just below the achingly beautiful Mt. Ritter and Banner Peak) was indeed “still” and healing for me. Escaping the sometimes painful travail of our profession is important and even essential at times.

Using the allegorical picture painted in Yeats’ poem makes sense to recognize Craig’s contributions to Houston Methodist Hospital, the Texas Medical Center (his adopted hometown), the NIH, FDA, his patients, colleagues, friends, and family. Think of Craig as Yeats’ idealistic fisherman. It may be a literal interpretation, but for me it directly connects to Craig’s efforts to improve healthcare practices and medical education over his 45-year career in Houston while seeking equanimity and stillness. It is something we all should be mindful of.

**Figure:**
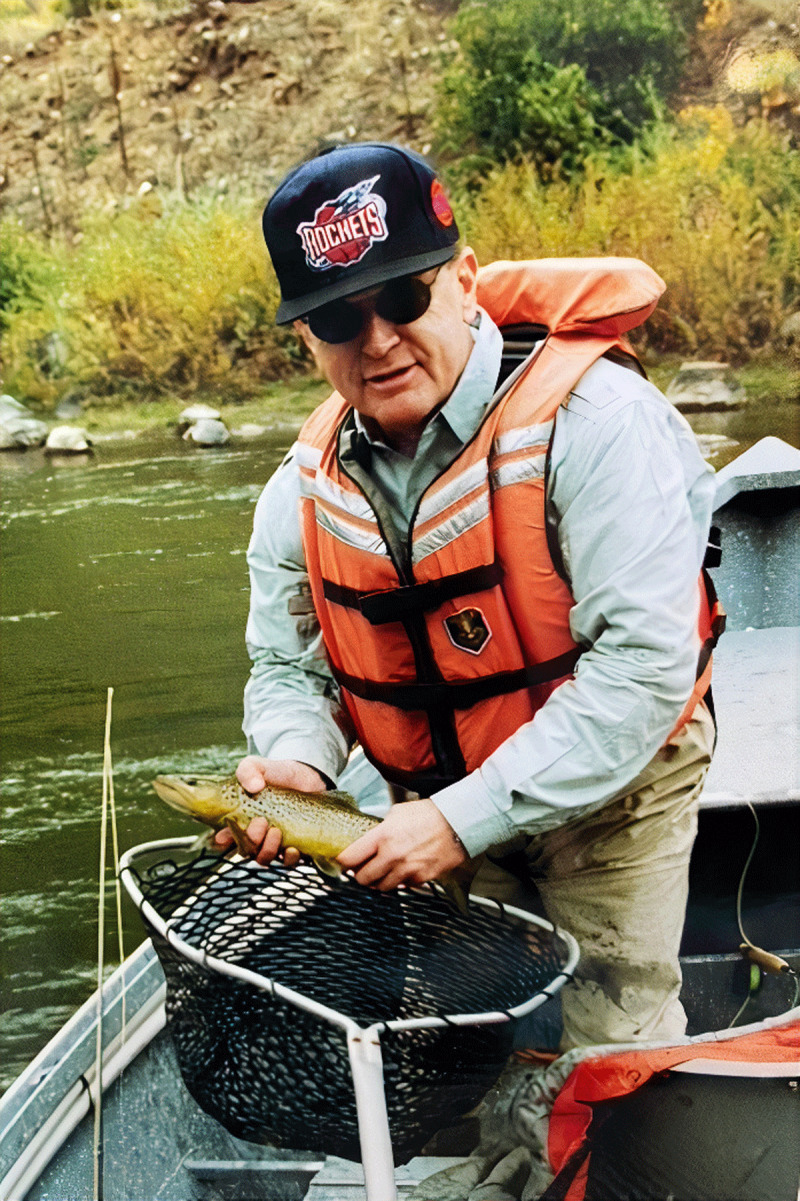
Dr. Craig M. Pratt, July 18 1945 – August 28 2021.

“The Fisherman” was published in 1916. It came after a tumultuous period in Yeats’ life, both politically and artistically. Yeats knew the sport, and he juxtaposed life’s frustrating events with more tranquil days spent fishing as a youth. We might think of Yeats casting gently from a water’s rocky outcropping to an inviting pool with hopes of a hit. That is the way I think of Craig and other avid trout fishermen: creating peace and tranquility in a hurly burly world with a flick of the rod, surrounded by nature’s wonder during dogged pursuit of an often-returned prize. “The Fisherman” was Yeats’ attempt to create the picture of a perfect man. Thus, the poetic image of the freckled man who goes to a gray place on the hill at dawn to cast his flies, which Yeats can still see.

As we emerge from the year’s challenges, we should seek stillness, peace, and tranquility in our own ways. Perhaps some “will climb up to a place where stone is dark with froth, and the down turn of [the] wrist when the flies drop in the stream” can be seen. Remembering how Dr. Pratt reveled in the stillness of those moments makes me smile as I reflect on his life and contributions.

